# Evaluation of Radiology Request Forms in a Tertiary Care Hospital: An Audit With a Focus on the Impact of Technological Intervention

**DOI:** 10.7759/cureus.13335

**Published:** 2021-02-14

**Authors:** Muhammad Danish Barakzai, Zara Za Sheer, Azeemuddin Muhammad, Amna Alvi, Noman Khan, Waseem M Nizamani, Madiha Beg, Saad Siddiqui

**Affiliations:** 1 Department of Radiology, Children's Hospital of Eastern Ontario, Ontario, CAN; 2 Department of Community Health Sciences, Aga Khan University, Karachi, PAK; 3 Department of Radiology, Aga Khan University, Karachi, PAK; 4 Department of Radiology, Prince Sultan Military Medical City, Riyadh, SAU; 5 Department of Radiology, Radiology Associates, Peshawar, PAK

**Keywords:** radiology, quality improvement, healthcare technology, artificial intelligence in radiology

## Abstract

Radiology request forms are the basis of communication between referring physicians and radiologists. These are the sole documents on the basis of which a justification to carry out a radiological procedure is carried out. However, across the globe, there is a problem of inadequately filled radiology request forms. Several interventions like standardization and the use of technology have been proposed worldwide to overcome the shortcomings of inadequately filled radiology request forms. We carried out a two-phase audit assessing the impact of a technological intervention on the quality of radiology requests with the results showing marked improvement in key parameters. A subset analysis was also done to highlight the importance of radiology request forms by following the patients' treatment course. The remaining shortcomings highlight the importance of training sessions and refresher courses for junior doctors in order to familiarize them with the importance of adequately filled radiology request forms.

## Introduction

Request forms for radiology are an important tool of communication used by hospitals and physicians to refer patients for radiological investigations. However, their importance is much underrated. In addition, there is a lack of standardization across different institutions when it comes to radiology request forms [1].

A radiology request form plays an important role in both diagnosis and treatment. These forms make the basis for performing radiological studies, which many a time includes the use of modalities with ionizing radiation. These are the sole documents on which justification to carry out an examination is performed [1].

The Royal College of Radiologists (UK) guideline states: “Requests should be completed accurately and legibly to avoid any misinterpretation; ideally, they should not be handwritten. Reasons for the request should be clearly stated, and sufficient clinical details should be supplied to enable the imaging specialist to understand the particular diagnostic or clinical problems to be resolved by the radiological investigation” [2].

Providing the correct biographic data of the patient and requesting a proper radiologic investigation in a timely manner is the responsibility of both the treating and request ordering physician [3-11].

Before accepting a request form, a radiologist should be aware of the clinical condition of the patient and the context of the requested examination prior to justifying it. It is, therefore, of utmost importance that radiological request forms be properly filled. The radiologist has the ultimate responsibility for justifying the requested examination and decision on the practical aspects of patient radiation exposure and the radiology request form is the basis for this decision. The problem of an inadequately completed radiology request form is considered widespread across the globe [8-11].

Objectives

To perform an audit for the adequacy of computed tomography (CT) request forms received at the radiology department of a tertiary care hospital in Pakistan in two phases and to assess the outcome of intervention via the implementation of an online system for radiology request form generation.

## Materials and methods

The audit was conducted in two phases. In the first phase, an audit was done of a period when forms were filled manually while in the second phase a change was brought into the system, which enabled the online generation of a radiology request form for emergency room and in-house patients.

For the first phase, radiology request forms of CT examination performed in the month of April 2013 were retrospectively assessed. For the second phase, re-audit was performed on similar parameters for radiology requests forms of CT examinations performed in April 2017 after a change of the online request generation system was implemented.

In phase I, 10 different fields in the request form were evaluated for completeness. These fields included: patient's mode of transport (i.e. ambulatory, wheelchair, or trolley), name of treating physician, complete patient identification (i.e. name and medical record number), patient location, ordering physician's name and contact number, examination required, date requested, clinical history, patient's risk of fall, and any known allergies.

In phase II, the new format (electronically generated online request forms) were assessed with the same method using the same parameters, and various fields on the request form were evaluated for completeness These fields again included: patient's mode of transport (i.e. ambulatory, wheelchair, or trolley), name of treating physician, complete patient identification (i.e. name and medical record number), patient location, ordering physician's name and contact number, examination required, date requested, clinical history, patient's risk of fall, and any known allergies.

Additional 150 request forms were evaluated in each phase according to the appropriateness of the investigation by assessing whether radiological diagnosis matched clinical question, whether the treatment of the patient continued according to the CT diagnosis, and hence retrospectively assessing the role played by an appropriately filled radiology request form for justification of the examination.

The study was performed at the department of radiology, Aga Khan University Hospital. The duration of the study was one month for each phase. A total of 935 request forms were reviewed in the first phase while 762 forms were evaluated in the second phase. Sampling was done by non-probability consecutive sampling. As the intervention was only done for the emergency room and in-house patients, therefore outpatient referrals to the department were excluded from the analysis. Data were entered and analyzed by using the Statistical Package for the Social Sciences (SPSS) statistical package version 19 software.

## Results

A total of 935 request forms were evaluated in phase I and 762 in phase II. In combination; the required information was adequately documented in 79.6 % of request forms in phase I and 96.3 % in phase II.

**Figure 1:**
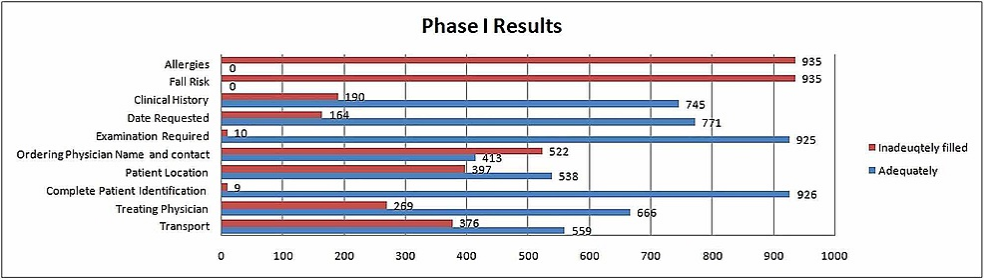
Phase I results

For phase I, only 59.7% of forms had an adequate mention for the mode of patient transportation. The name of the treating physician was given on 71.2% forms. In 57.5 % of forms, the patient location was filled. The ordering physician's name and contact number were given in 44.1% and 46.7% forms, respectively. In 98.9%, forms the field of examination required was filled. The date requested was mentioned in 83.4% of the forms. Clinical history was provided in 79.7%, and none of the forms mentioned any known allergies (Figure 1).

**Figure 2:**
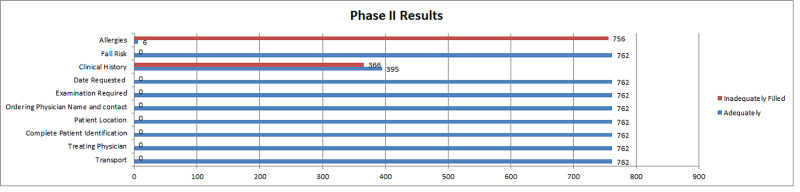
Phase II results

For phase II, fields with 100% completion were names of treating physician, ordering physician, contact information, date requested, patient identification, visit, transport, exam order, and patient’s risk of fall. It is of note that all of these parameters were automatically generated due to the online nature of the form. In the rest of the parameters, only 0.78 % of the forms mentioned the allergies, a brief clinical history was filled in 96.3 %, the provisional diagnosis was written in 51.9% of forms, and any special instructions were mentioned in 70.2% of forms (Figure 2).

Subset analysis for additional questions in order to retrospectively assess the justification of procedure was also performed for both phases. The final radiological diagnosis matched the provisional clinical diagnosis in 56.8% of cases in phase I and 77% in phase II. The treatment and diagnosis were continued according to the radiological conclusion for 87.9% in phase I and 91.1% in phase II. The CT examination was justified according to the clinical diagnosis in 91.3% of phase I and 85.9% of cases in phase II.

## Discussion

The radiology request form is a document of immense importance with medicolegal standing. It ensures that the correct procedure is performed on the correct patient, the procedure, which often involves the use of ionizing radiation, is justified, and the radiology staff are aware of any special circumstances like known allergies and fall risk. Most of these parameters also fall under the core of International Patient Safety Goals (IPSGs) [12].

Across the world, radiologists face problems with inadequately filled or incomplete radiology request forms, which make the justification of the procedure a difficult task. A number of interventions have been proposed like the standardization of forms in countries with nationwide health services or the use of technology as was the case in this audit [1-4].

From the results of this audit that there is marked improvement in phase II results as parameters related to IPSGs on radiology request forms as most of the parameters are automatically filled by the system for the selected patient. In addition, due to the linking of the system with medical records, the system automatically updates parameters like fall risk from updated nursing notes.

The important clinical information regarding provisional diagnosis was lagging behind the expected standards in both phases. This highlights the importance of special training of junior doctors who are mostly filling up the request forms regarding its importance and the impact it carries on overall patient care. The subset analyses in both phases show that the treatment of the patient was either continued or modified according to results of the radiological study, reinforcing the importance of adequately filled radiology request forms, which, in turn, enable the radiologist to analyze the scan in lieu of clinical question and history of the patient [9,13].

## Conclusions

This two-cycle audit shows marked improvement in the adequacy of radiology request forms after intervention by the use of technology. The results reinforce that standardization and technological interventions are needed in order to improve the quality of radiology request forms. The remaining deficiencies highlight the importance of orientation and refresher courses on the importance of radiology request forms to junior doctors of referring teams. It is proposed that a session on this aspect be incorporated in the orientation program of health facilities at the time of new inductions.
